# Fertility sparing treatment in borderline ovarian tumours

**DOI:** 10.3332/ecancer.2015.507

**Published:** 2015-02-03

**Authors:** Rosa Maria Alvarez, Daniel Vazquez-Vicente

**Affiliations:** 1 Department of Gynaecological Oncology, St Bartholomew’s Hospital, London EC1A 7BE, UK; 2 Gynaecological Oncology Unit, Fundacion Jimenez Diaz, Madrid 28040, Spain

**Keywords:** borderline ovarian tumours, fertility-sparing surgery, conservative treatment

## Abstract

Borderline ovarian tumours are low malignant potential tumours. They represent 10–15% of all epithelial ovarian malignancies. Patients with this type of tumour are younger at the time of diagnosis than patients with invasive ovarian cancer. Most of them are diagnosed in the early stages and have an excellent prognosis. It has been quite clearly established that the majority of borderline ovarian tumours should be managed with surgery alone. Because a high proportion of women with this malignancy are young and the prognosis is excellent, the preservation of fertility is an important issue in the management of these tumours. In this systemic review of the literature, we have evaluated in-depth oncological safety and reproductive outcomes in women with borderline ovarian tumours treated with fertility-sparing surgery, reviewing the indications, benefits, and disadvantages of each type of conservative surgery, as well as new alternative options to surgery to preserve fertility.

## Introduction

Borderline ovarian tumours (BOTs), or low malignant potential tumours, represent 10–15% of all epithelial ovarian malignancies [1]. Their incidence is low, and is calculated in European series at around 4.8/100,000 new cases per year, and even lower in American series, 1.5–2.5/100,000 cases per year [2]. Patients with BOTs are younger at the time of diagnosis than patients with invasive ovarian cancer (IOC), they occur in women at approximately 40 years of age, but in 27–36% of cases the tumours occur at a younger age [1, 3].

BOTs are classified according to the FIGO classification used for ovarian tumours [3, 4]. The majority of BOTs are diagnosed in the early stages, 70–80% are diagnosed at stage I, compared with 25% of carcinomas [5], about 20% present in advanced stages. Ten years overall survival rate for those in the initial stages is 90%, and 60–70% of those in the advanced stages [6]. The survival rate in patients with BOTs in stage IA is 98–100% [3]. The main prognostic factors are the FIGO stage and type of peritoneal implants (with or without invasion) [1, 7].

BOTs are characterized by the absence of stromal invasion. Histologically, BOTs are characterized by cellular proliferation, stratification of the epithelial lining of the papillae, nuclear atypia, and mitotic activity [1, 8, 9]. There are different histological subtypes, most of them are serious tumours, about 53–65%. Mucinous BOTs constitute between 32% and 42% of the total. The rest of the BOTs (less than 5%) are composed of endometrial tumours, clear-cell tumours, Brenner’s tumours, and other histology [2, 3].

The diagnosis of BOTs is based on histological examination, with normal or only modestly increased of CA-125 level, no free fluid in the pelvic cavity and no other features of malignancy [10]. The clinical presentation, treatment and prognosis of BOTs differ drastically from the invasive carcinoma. Some patients with BOT (16–30%) are asymptomatic when diagnosed and the discovery is incidental; nevertheless, when there are symptoms these are often non-specific, such as pelvic pain or abdominal distension [3, 8].

It has been quite clearly established that the majority of BOTs should be managed with surgery alone. Because a high proportion of women with BOTs are young and the prognosis is excellent, the preservation of fertility is an important issue in the management of these tumours [1, 11]. The management and prognosis of advanced-stage BOTs, however, have not been clearly established [12].

In this systemic review of the literature, we have evaluated the oncological safety and reproductive outcomes in women with BOTs treated with fertility-sparing surgery, as well as new controversies in BOT management and alternative options to surgery to preserve fertility.

## Methods

An electronic database search (EMBASE, MEDLINE, PubMed) was performed with the objective of identifying all studies investigating BOT, published up to August 2014. No date restrictions were placed; relevant citations were hand searched.

The following medical terms were searched: borderline tumour, ovarian, ovary, low malignant potential, conservative surgery, fertility-sparing surgery, laparoscopy, invasive implants, non-invasive implants, micropapillary patterns, microinvasion, advanced stages, recurrence, survival, mortality, pregnancy rate, fertility treatment, assisted reproductive technique, ovarian stimulation, in-vitro fertilization, cryopreservation.

Early and advanced stages of serous and mucinous borderline ovarian tumours have been included in this review, excluding rare entities because of their low incidence.

All pertinent articles were retrieved and the relative reference lists were systematically reviewed in order to identify additional studies that could be included. All original studies, meta-analyses, systematic reviews and case reports published in English were considered. The prevalence of recurrence, death and pregnancy was calculated for each study.

## Fertility sparing treatment

Patients with BOT are younger than patients with invasive ovarian cancer, 27–54% are younger than 40 years, and many of these patients wish to preserve fertility [8, 13]. Management of BOTs has changed from radical surgery to a more conservative treatment, but there is still a lot of debate regarding the extension of the surgical procedure. Fertility-sparing surgery is considered when the uterus and ovarian tissue in one or both ovaries are preserved. There are two types of fertility-sparing surgeries, unilateral salpingo-oophorectomy (USO) and unilateral ovarian cystectomy with or without contralateral ovarian cystectomy. The standard radical surgery is hysterectomy and bilateral salpingo-oophorectomy (BSO) [14].

### Cystectomy and unilateral salpingo-oophorectomy

Fertility-sparing surgery is the standard treatment in young patients with early stage BOTs. Ovarian cystectomy provides a better opportunity for fertility preservation than adnexectomy because of the removal of less ovarian tissue. Many studies have suggested that bilateral cystectomy is associated with an increase in recurrence rate, because of the risk of some malignant cells may be left in situ [15, 16]. Palomba *et al* (2007), in a prospective randomized controlled trial, showed no significant difference between bilateral cystectomy and USO plus contralateral cystectomy [15].

Published data on the oncologic outcomes of cystectomies are limited, and these studies have included a small number of patients. In most of the studies, the recurrence rate in the fertility-sparing surgery does not differ significantly by type of surgery, therefore, cystectomy can be considered for patients with bilateral tumours or previous USO [13, 15, 17]. In other studies, the rate of recurrence tends to be higher following cystectomy than USO. The rate of recurrence after cystectomy varies from 12–36.3% [18, 19, 20]. Boran *et al* (2005) reported a recurrence rate after cystectomy of 15% and 2.4% after USO, but it was not statistically significant [21]. Song *et al*’s study (2011), comparing cystectomy with USO, showed a recurrence rate of 13.9% in the cystectomy group and 6.0% in the USO group, but this difference was not statistically significant [1]. Another study performed by Lim-Tan *et al* (1988) has recommended histologic analysis of the margins after cystectomy, to reduce the risk of recurrence, as they found that presence of tumour at the surgical margin was a risk factor for ovarian recurrence [16], however, this practice does not modify the management of BOTs. Several series published of oncologic outcomes after conservative treatment are summarized in Table 1.

The rate of recurrence is generally increased in the fertility-sparing approach, between 12 and 58%, compared with 5% in radical surgery [5, 22–26]. However, numerous studies have demonstrated the safety of conservative surgery, with favourable prognosis and comparable survival rate for conservative or radical surgery [5, 27, 28]. The largest reported prospective series of BOT by Zanetta *et al* (2001) showed that, although the risk of recurrence is higher after conservative surgery (18.5%) than after radical surgery (4.7%), all but one patient with borderline lesion recurrence after conservative surgery were salvaged [5]. Yinon *et al* (2007) revealed no significant difference between cystectomy and USO in mean recurrence rate (22.7% for the cystectomy group and 27.5% for the USO group) [17]. In contrast, Morice *et al* (2001) found different outcomes, with higher recurrence rate in cystectomy than USO (36.3% versus 15.1%, respectively) [19].

Ovarian cystectomy can elevate the risk of cyst rupture and intra-abdominal spillage in 70%, which is associated with an increase of relapses, and contamination of the abdominal wall [20]. Poncelet *et al* (2006) found a higher rate of rupture in laparoscopic surgery compared to in laparotomic surgery [29]. However, there are no prospective studies on BOTs on this issue and its prognostic factor value, spilling should be avoided until otherwise confirmed. As recently demonstrated by some authors, these risks can be reduced to an acceptable level by the systematic use of an endoscopic bag and abundant copious peritoneal washing [20, 30]. Seracchioli *et al* (2001), reported 19 patients with BOT who were treated by a conservative laparoscopic approach: 11 had cystectomy and 8 had USO. Among the patients who had unilateral tumours, the ovarian biopsy of the contralateral ovary did not show malignity. Spillage occurred in two cases. One patient developed a local recurrence in the same ovary within 6 months. Cystectomy was performed at the first and the second surgical treatment. They did not find any relation between rupture and tumour recurrence, which was in accordance with other studies [30]. The recurrence rate after laparoscopic surgery in the literature is similar to that documented after the abdominal approach, which confirmed that conservative laparoscopic treatment is as effective as laparotomy [16, 24, 30, 31].

The higher risk of local relapses found in conservative surgery does not exert a statistical impact on the invasive recurrence rate because most of the recurrences are borderline lesions, and this is not associated with decreased overall survival. Recurrence is normally related to local residual tumour in the ipsilateral ovary after cystectomy rather than tumour localization in the contralateral ovary. Special attention should be paid to the remaining ovary after conservative surgery. Recurrent disease can be detected with close follow-up and can be treated accordingly [5]. For BOTs affecting both ovaries without healthy ovarian tissue, BSO should be performed. However, in certain cases, incomplete resection of one ovary can be considered, when a young patient still has a desire for childbearing, and the same precautions as for simple cystectomy apply [20].

Safety of conservative treatment has been confirmed and even expanded to include women with advanced-stage disease. For patients with stage II, III, or IV BOTs who have not completed childbearing, conservative surgery could be an option [32].

To conclude, USO may provide a safe therapeutic treatment for BOTs in women wishing to preserve fertility, and should be considered as the first choice of fertility-sparing treatment and for massive involvement of the ovarian parenchyma when it is not possible to preserve healthy ovarian tissue, and ovarian cystectomy should be considered for women with one ovary or with bilateral tumours who wish to preserve their childbearing potential, and who are willing to undergo careful and prolonged follow-up examinations [13, 14, 27, 33].

### Surgical staging

There is no standardized surgical treatment for patients with BOTs, but the guidelines for surgical treatment of BOT are similar to those for ovarian cancer and, in women who have fulfilled their reproductive wishes include exploration of the abdominal cavity, total hysterectomy with BSO, inframesocolic omentectomy, resection of macroscopically suspicious lesions, and peritoneal washing [32]. Multiple peritoneal biopsies is in disuse due to its low sensitivity where no suspicious lesions are present [3, 34].

Pelvic and paraaortic lymphadenectomy is not considered necessary, because there is no significant improvement in recurrences rates [3, 34]. The lymph node involvement does not decrease survival. Lymphatic involvement, despite having no prognostic value in BOTs, recurrence or progression to carcinoma in this area is exceptional and therefore does not justify the morbidity associated with systematic lymphadenectomy. Other authors have reported that the absence of surgical staging in patients with stage I BOT does not modify survival, even if the recurrence rate is increased [13, 19]. Longacre *et al* (2005) detected lymph node involvement in 29% of patients who underwent a staging procedure. In this study, 17.6% of extraovarian recurrences and 10% of transformations to carcinoma occurred in lymph nodes, but they have found no association between nodal involvement and disease recurrence or overall survival [26]. Leake *et al* (1992) found that nodal involvement by borderline lesion did not significantly affect survival but was associated with a higher rate of recurrence [35].

Systematic restaging operation is controversial due to its minor effect on clinical management and outcome. The main reasons for nonsystematic initial surgical staging in BOT are on one hand that most surgeries are performed due to a suspicious benign cyst [36] and, on the other hand, the potential sampling error of intraoperative frozen section [37]. Du Bois *et al* (2013) performed a re-staging operation in 44.3% of cases and they found a significant improvement in the prognosis [38]. A systematic review on re-staging procedures in BOT, performed by Ness *et al* (2002), reported upstaging in 17.6% and residual tumour in 24.7% [39]. However, these findings regarding a prognostic impact of re-staging were controversial. Ipsilateral salpingo-oophorectomy, especially after cyst rupture, could be considered [40], but always recommending a case-by-case approach, taking into account the adequacy of initial surgery and the tumour subtype [29, 41]. If a simple cystectomy has been performed for a supposed benign cyst and there is an incidental discovery of a BOT, no further surgical procedure is generally needed if complete exploration of the abdominal cavity has been performed; no spillage occurred during surgery; and the borderline lesion is on the inner side of the cyst without vegetation on the outer side of the cyst. Then very close follow-up should be enough to detect recurrent disease [3, 20].

BOTs are bilateral in 25–50% of patients with serous type tumours and in 5–10% of patients with mucinous type tumours [9, 42]. There is controversy on routine ovarian biopsy of the contralateral ovary in Stage IA. Some authors propose systematic biopsy of the contralateral normal-appearing ovary in cases of unilateral disease in order to evaluate the presence of unapparent microscopic borderline tumour. However, the detection of a small focus of borderline disease in a macroscopically normal-appearing ovary with this procedure is very low [19, 43]. Furthermore, several series have reported recurrences in patients who had a normal biopsy of the contralateral ovary [24, 44]. Other authors do not suggest systematic biopsy due to this is not a procedure without side effects as it may induce infertility of a mechanical nature because of post-operative ovarian adhesions [19, 21, 45], reported to be approximately 14% [44].

Morice *et al* (2001) performed a biopsy on the contralateral ovary in 14 patients, and none of these biopsies was positive [19]. This was also reported by Tazelaar *et al* (1985) [24]. Boran *et al* (2005) performed 15 contralateral ovarian biopsies and one of them was positive for a small neoplastic focus of BOT [21]. Park *et al* (2009) reported 22 patients who underwent biopsy of the normal-appearing contralateral ovary, none showed tumour. In contrast, 11 of the 22 patients who underwent cystectomies to remove benign-appearing cysts of the contralateral ovary had borderline lesions [8]. Biopsies of suspicious lesions are indicated, but there is no evidence that routine biopsies of contralateral ovary can exclude recurrent disease in all cases [19, 21, 43]. Careful macroscopic inspection of the normal ovary should be enough [19].

Appendicectomy can be performed for mucinous BOT, but in cases of isolated tumour with normal appearing appendix systematic appendectomy is not obligatory [28]. Park *et al* (2009) performed appendectomy in 63 of 245 patients (25.7%) with mucinous BOT, but none revealed appendiceal involvement [8].

### Sparing in advanced stage

Most of the reports have not specifically focused on advanced-stage BOTs [12]. For patients with advanced-stage disease, the clinical approach is not clear and fertility-sparing surgery is not usually accepted [32]. Camatte *et al* (2002) reported 17 women with stage II or III BOT, treated with fertility-preserving surgery, only two of them recurred, and there were no deaths at a median follow-up time of 60 months [46].

The most important prognostic factor in BOTs is the type of implants, invasive or non-invasive, as it is well known that the prognosis of patients with invasive implants is much worse [12]. Advanced-stage BOTs with non-invasive implants can be safely treated with conservative surgery. For patients with invasive implants fertility-sparing surgery could be considered, but with an individualized approach [12, 25].

### Sparing in recurrence

The most common site of recurrence in BOTs after conservative surgery is the remaining ovary. In these cases, the recurrence can be safely treated with surgery, and fertility-sparing surgery can be performed if the patient has a strong desire to become pregnant [1, 19]. BOTs with invasive implants tend to relapse more frequently than BOTs with non-invasive implants (40% and 10% respectively; [12]). Furthermore, in patients who initially had tumours with non-invasive implants, when recurrence develops after the primary treatment, most of the relapses have a borderline histology [5, 15–17]. Uzan *et al* (2013) reported conservative surgery for the first recurrence in 26 patients (68%) of the 38 relapses observed after conservative treatment for stage I serous BOT [47]. Cheng *et al* (2009) presented six patients with recurrent BOT who had fertility-sparing surgery. Five patients retained normal menstrual function. Three women had successful pregnancies, with a spontaneous pregnancy rate of 50%. One of the six patients developed recurrence once more. However no disease-related deaths occurred [48].

Conservative surgical treatment for recurrence in the ipsilateral ovary would be indicated for women younger than 40 years old who want to preserve their fertility, who are committed to exhaustive follow-up, and without invasive implants, and radical, for patients older than 40 years old, with their childbearing desires completed, would find it difficult to adhere to follow-up requirements, or with invasive implants. When an extra-ovarian borderline or invasive relapse occurs, cytoreductive surgery should be performed [3, 49]. The optimum cytoreductive surgery is an independent prognostic factor, and will determine the over survival. Crispens *et al* (2002) reported a mortality rate in patients with optimal debulking of 12%, compared to 60% of those who were suboptimally debulked [50].

The spontaneous pregnancy rate after the second conservative surgery in cases of recurrent disease is satisfactory, therefore this procedure should still be considered for young women who desire preservation of fertility, but careful follow-up is needed to detect a new recurrence.

### Indications for completion surgery

The management of patients treated with conservative surgery after childbearing is not clear. Some authors have suggested definitive surgery to remove the contralateral ovary after successful pregnancies [32, 42]. Another option would be delaying definitive surgery until recurrence, taking in account that recurrent disease after conservative surgery is borderline in most cases, and the most common recurrence site is the remaining ovary and could be successfully treated with surgery, or until menopause, to preserve the endocrine function.

Histological criteria is the most important risk factor of recurrence, particularly the invasive form, justifying completion surgery [30]. Morice *et al* (2012) estimated a mean time to progression to carcinoma of 75 months for serous BOT and 33 months for mucinous BOT, with a progression rate to invasive cancer of 2–3% [2]. Kurman *et al* (1993) have estimated malignant transformation in 0.75% of ovarian serous BOT [51]. Gershenson *et al* (1998) found that approximately 30% of patients with serous BOT with non-invasive implants would develop progressive or recurrent tumours. They observed that the presence of macroscopic residual tissue was a predictor of disease-free survival [52].

The rate of recurrence in patients with invasive implants is 38% [53–55]. Morice *et al* (2012) showed that in cases of serous BOTs with microinvasion the risk of recurrence was 15%, of which 35% were invasive disease, with a death from the disease of 6% [2]. For patients with non-invasive implants most recurrences occur within the first 5 years after diagnosis, but this does not provide evidence of an indication for completion surgery, seeing that survival is not affected as the borderline recurrent disease can be treated with curative surgery [5, 56, 57]. Daraï *et al* (2013) have recommended completion surgery after childbearing in cases of microinvasion or invasive peritoneal implants [58]. In cases of mucinous BOT with intraepithelial carcinoma completion surgery has been recommended if cystectomy has been previously performed. In conclusion, only microinvasion or intraepithelial carcinoma would be indications for completion surgery.

## Preoperative diagnosis

The final diagnosis of an ovarian tumour is based on the histopathological study, but some tumour markers and imaging techniques have been proposed for the preoperative diagnosis of BOT. It is important to differentiate the nature of the ovarian mass prior to surgery in order to decide if surgery is required and the appropriate type of surgery [59].

### Symptoms

Women with BOT are asymptomatic more frequently than patients with IOC. The symptoms are very unspecific; therefore, it is not possible to orientate the diagnosis of an ovarian mass based on the symptoms only [60]. Around 80% of patients present with abdominal symptoms as abdominal distension, abdominal pain or increased abdominal size, about 15% have gynaecological symptoms, 10–35% gastrointestinal symptoms, 5–26% urinary symptoms, 5–7% general malaise and weight loss [60, 61].

### Tumour markers

Several tumour markers have been studied in this field. The most outstanding is CA 125, which is elevated in 24–61% patients with BOT [62–64]. The mean serum CA 125 concentration is lower in BOT than in patients with invasive cancer, with a median serum level of 34.7 U/ml and 401.5 U/ml, repectively [65], and higher in advanced-stage BOT than in early stage [57, 62–64].

Other tumours markers studied for diagnosis of BOT, as CA 19.9, Tissue polypeptide antigen, Carcinoembryonic antigen, Endoglin and Tetranectin have not demonstrated be useful. Other serum markers such as calprotectin, Oviductal glycoprotein 1 and Growth differentiation factor-15 may be useful for diagnosing BOTs. However, more studies are needed to confirm the application of these markers in clinical practice [63, 66].

### Imaging techniques

**Ultrasonography:** In patients with adnexal masses transvaginal ultrasound is the primary screening imaging technique. The most frequent imaging in BOT is a cyst with internal papillae and septae, observed in 49–63% of BOTs [67, 68]; around 18% of BOTs present multiple septa [67]. But these features are not highly sensitive markers of BOT. Borderline lesions can appear as unilocular cyst without endophytic papillary growth up to 17% [64, 67, 68]. Serous BOT present with internal solid parts or papillary pattern in 78%, and mucinous BOT in only 40% of cases [64, 67]. Ultrasound is usefull to differenciate between benign and malignant tumours, but it is very difficult to differenciate between BOT and benign lesions.

**Magnetic resonance imaging (MRI):** BOT can appear with different findings on MRI, such as unilocular cysts, cysts with papillary projections, septate lesions with excrescences or solid lesions with exophytic papillary projections [69]. The solid components have normally intermediate signal intensity [70]. The radiological appearance of BOT is similar to those of early stage ovarian cancers [70], and there are not adequate prospective studies in the literature that have estimated the accuracy of MRI in discriminating BOT from benign or malignant lesions. Therefore, MRI appearance of BOT does not seem to allow an accurate preoperative diagnosis. A recent systematic review showed that MRI has sensitivity of 92% and specificity of 85% for the detection of borderline or IOC [71].

**Computerized tomography (CT):** CT is normally used for the detection of extrapelvic disease and to estimate the stage of the disease, but it is not very useful for differentiation between BOT and benign or malignant lesions. [70].

**Positron emission tomography (PET):** It is well known that benign cysts and BOT have a different distribution pattern of the metabolic tracer when comparing with invasive cancer [59, 72]. The combination of the functional imaging provided by PET plus the morphologic appearance of the tumour on MRI, appears to increase the capacity of discrimination between borderline and malignant lesions [59]. Therefore, the presence of complex characteristics on MRI plus a benign pattern on PET suggest BOT, nevertheless these should be corroborated by bigger sample studies.

**Combined positron emission tomography/computerized tomography (PET/CT):** The PET/CT discrimination capability between BOT and benign tumours had been compared recently with ultrasonography, MRI and CT, showing a higher accuracy of PET/CT [73]. Nonetheless in a previous study PET/CT was not useful when differentiating between both diagnoses [74], so again more studies are needed to clarify this issue.

The combination of various diagnostic modalities, mostly tumour markers and ultrasonography, might increase the accuracy of preoperative diagnosis of BOT, but the final diagnosis requires surgical excision and histological examination of the tumour.

## Intraoperative diagnosis. Role of frozen section

Intraoperative histological diagnosis of BOT should be obtained by frozen section, even when it is known that frozen section has a potential sampling error [44]. In cases of unilateral and suspicious of benign adnexal masses, ovarian cystectomy is normally performed. Adnexectomy would be indicated when no normal ovarian tissue is adjacent to the mass. In both cases the contralateral ovary should be carefully inspected and the specimen should be sent for frozen section examination [21]. When the mass is bilateral the most conservative approach would be the best option, with bilateral cystectomy when possible or oophorectomy plus contralateral cystectomy, and sent for frozen section examination [21]. Intraoperative frozen section can help to decide if surgical staging shoud be performed [59].

## Follow-up

Most studies have demonstrated that the risk of recurrence is based on histology and initial stage, but also on CA 125 serum level and the type of conservative surgery performed [75]. Nonetheless, there is no protocol or score to assess the individual risk of recurrence. A close follow-up is recommended at least for the first two years after diagnosis, as previous studies have reported that most BOT recur during this period [76]. Given the absence of consensus, a systematic visit every 6 months for the initial period has been proposed, with a long follow-up of at least 10 years, at least once a year, especially for patients with advanced stages [2]. The follow-up should include a physical examination, ultrasonography and tumour markers evaluation [5].

## Oncologic outcomes

Several studies have compared the oncological outcomes of fertility-sparing surgery (Table 1). From an oncological point of view, there are many studies in the literature that show that the recurrence rate in patients who underwent conservative surgery is significantly higher (6.5–29.5%) than in patients who underwent radical surgery (0–8%) [5, 8, 19, 40, 42]. Palomba *et al* (2010) found an acceptable rate of recurrences for ultra-conservative surgery [33]. The median time to recurrence has been reported to be 5–7 years by some authors [12, 50]. 

In the study of Song *et al* (2011), the rate of recurrence was higher in the fertility-sparing surgery group (7.7%) than in the radical surgery group (4.9%), with a median follow-up time of 56 months, however, this difference was not statistically significant. They found a 5-year recurrence-free survival rate in the radical surgery group of 95.2% and in the conservative surgery group of 93.8% [1]. Of the 19 patients who relapsed, all patients apart from one underwent secondary surgery with or without adjuvant chemotherapy. The rate of recurrence did not differ significantly between USO and ovarian cystectomy (5.9% versus 13.2%, respectively). The site of recurrence in all patients was the remaining ovary. Stage, presence of invasive implants, and micropapillary pattern were significant prognostic factors [1]. Park *et al* (2009) showed a recurrence rate somewhat higher in the fertility-sparing surgery group than in the radical surgery group. The mean interval from initial surgery to recurrence was 45 months [8].

The reported recurrence rates in the literature after radical or fertility-sparing surgery for advanced-stage BOTs vary (5.4–30.8% versus 23.8–66.7%, respectively) [12]. Song *et al* (2011) in their study on advanced-stage BOTs, found a recurrence rate of 15% after radical surgery and 20% after fertility-sparing surgery. The median follow-up time was 71.4 months. Four patients relapsed in a median interval of 40 months after the primary treatment; two of them had a recurrence as an IOC [12].

Tsai *et al* (2011) found a recurrence rate of 22.6% in women who underwent conservative surgery; all patients who had a relapse were alive and disease-free after 36 months of follow-up from the recurrence [27]. Zanetta *et al* (2001) found a recurrence rate higher for women undergoing fertility-sparing surgery (18.5%) compared with radical surgery (4.7%), and a recurrence rate after conservative and radical surgery for stage I disease was 15.2% and 2.5%, respectively, and 40% and 12.9% for advanced stage [5]. Patients younger than 40 years have a more favourable prognosis with a 5-years survival rate of 99%. At the age of 70, the 5-year survival rate drops to 85%, probably in relation to the greater comorbidity related to the surgery [3].

Invasive recurrent disease is a rare event after conservative treatment. Most recurrences are borderline lesions that can be treated by curative surgery without any impact on survival [5, 8]. Zanetta *et al* (2001), reported a series of 189 patients who underwent fertilitysparing surgery, with seven cases of invasive recurrence, six of them were still alive after the treatment of their recurrence at the time of the publication [5]. Park *et al* (2009) observed nine recurrences in their series of 164 patients treated conservatively, only one was invasive [8]. Nevertheless, as these studies are retrospective, it is not possible to affirm that there is no potential impact on oncological safety after conservative treatment of borderline tumours [58].

In the case of patients with peritoneal implants conservative management may be an option if the implants can be completely removed [5, 57]. The only situation where recurrent disease could affect the prognosis is when the nature of the recurrent disease is invasive. Taking into consideration the poorer prognosis of BOT with invasive peritoneal implants, fertility-sparing surgery should be approached with caution in this setting [57]. The risk of progression to invasive carcinoma in the particular case of initial conservative surgery is 2–3% [2]. The risk of lethal recurrence in early stage is 0.5%, in patients with advanced stage increased to 2% [58]. Recent data suggest that mucinous BOT recur in the form of invasive carcinoma more often than serous BOT [77].

Tumour stage, progression to low-grade serous carcinoma, residual disease and the presence of invasive implants have been associated with prognosis [35, 36, 38, 52]. Du Bois *et al* (2013) performed a multicenter study with 1236 patients, representing the largest series of BOT about the analysis of prognostic factors [38]. The overall rate of invasive relapses was 2.3% and occurred in 30% of all relapses. Most relapses happen in the remaining ovary. Higher FIGO stages appear to be associated with higher recurrence rates [36, 38, 78].

Microinvasion and micropapillary growth pattern have been reported as independent prognostic factors by some authors [36, 75] but not by others. Du Bois *et al* (2013) found no prognostic impact of microinvasion or micropapillary growth pattern. They confirmed that the presence of peritoneal implants is an important predictor of recurrence and malignant transformation. However, the difference between invasive and non-invasive implants was not statistically significant, probably due to limited number of patients [38]. In another important study, performed by Longacre *et al* (2005), micropapillary BOT were also more frequently associated with invasive implants and decreased survival, but micropapillary growth did not have a significant adverse effect on survival when controlled for implant type [26].

Longacre *et al* (2005), in their series with 276 patients, found an overall survival and disease-free survival of 95% and 78%, respectively, 5% of patients died of their disease and an additional 6% had persistent disease at the last follow-up. Of the 74 patients who had a conservative surgery, 26% experienced a recurrence, and 20% of the recurrences occurring 5 or more years after initial diagnosis. In this study, invasive extraovarian implants were strongly associated with adverse outcome in patients with high-stage disease [26]. Similar results have been reached by some, but not all, investigators [25, 52, 55]. In this series, 12.4% of patients with advanced-stage disease had invasive implants, 50% of them died of their disease or had progressive disease, and only 10% of patients with noninvasive implants. In this study, 6–7% of patients with BOT developed transformation to low-grade serous carcinoma many years after the initial diagnosis [26]. Implant status is currently one of the most significant prognostic factors for advanced-stage BOT. The presence of residual disease was also associated with poor outcome in this series. Given the prolonged interval that may occur between initial diagnosis of BOT and the development of carcinoma, some authors have questioned whether transformation to carcinoma represent a true progression of BOT or a new independent primary peritoneal tumour [51].

To conclude, the presence of invasive implants or stromal microinvasion in the primary tumour increases the risk of disease progression, but taken individually these features are neither sensitive nor specific predictors of adverse outcome. These data suggest that the relative risk of disease progression in patients with BOT is a combination of clinical and pathologic features, including stage of disease, extraovarian implant status, stromal microinvasion and micropapillary growth.

## Reproductive outcomes

According to the literature, the rate of spontaneous pregnancy in BOTs treated with conservative surgery varies between 32 and 65% [27, 79]. Table 2 summarizes reported series comparing pregnancy outcomes after fertility-sparing surgery. The pooled estimate for spontaneous pregnancies in the systematic review performed by Darai *et al* (2013) was 54% [58]. In patients with advanced stage BOT, the spontaneous pregnancy rate is lower (34%) [58]. Song *et al* (2011) reported 155 patients with BOT treated with fertility-sparing surgery, with a pregnancy rate of 88.2% [1]. They found a pregnancy rate of 89.2% in the USO group and 85.7% in the cystectomy group [13]. Park *et al* (2009) reported that of 31 patients who had attempted to conceive, 27 patients had succeeded (32 single pregnancies and one twin pregnancy). To date, none of these patients has undergone radical surgery after completion of childbearing [8].

Song *et al* (2011) reported the reproductive outcomes in patients with advanced-stage BOTs. Five women with advanced-stage disease underwent fertility-sparing surgery (20%). All patients had regular menstrual cycles after the conservative surgery. Four of them had attempted to conceive; four spontaneous single pregnancies occurred and one patient conceived after ovulation induction. One patient who had invasive implants underwent radical surgery after completion of childbearing without any suspicious of recurrence. The date range between treatment and pregnancy was 15 months. Four healthy babies had been born and one patient was still in the second trimester of pregnancy at the time of the analysis [12].

Palomba *et al* (2007 and 2010) demonstrated that the use of cystectomy improves fertility results, particularly in patients at high risk of bilateral tumour [15, 33]. They published in 2007 the reproductive effectiveness of ultra-conservative surgery followed by 12 months of ovulation monitoring and timed intercourse. The aim of this study was to evaluate the balance between fertility and risk of recurrence of young patients with stage I bilateral BOT who desire to conceive. They concluded that the ultra-conservative surgery has significant reproductive advantages over the standard procedure, in terms of cumulative pregnancy rate and time to conceive [15].

The conservative treatment of bilateral BOTs has advantages through the preservation on a greater amount of ovarian tissue. Palomba *et al* (2010) showed no significant variation of serum FSH levels during the 11-year follow-up after bilateral cystectomy. Patients who underwent ultra-conservative surgery had better response to ovarian stimulation in IVF cycles, showing less cancelled cycles, shorter stimulation time and use of lower dose of gonadotrophins. This study demonstrated that in well-selected patients (women without history of infertility, younger than 35 years old and with basal FSH lower than 15 IU/l) with bilateral stage I BOT, ultra-conservative fertility-sparing approach followed by a fertility programme gives real reproductive advantages. The higher risk of recurrence of the bilateral cystectomy could be managed with a close follow-up and completion surgery after childbearing [33].

Another factor to consider is the histological subtype of the borderline tumour. In some studies the fertility results were better in patients with non-serous compared with serous BOT [80, 81]. Further studies are required to clarify the role of the antral follicle count and AMH serum levels in patients with BOT, which are known to be potential predictive factors of ovarian reserve.

## Alternative options to preserve fertility

Conservative management and therapeutic alternative options for fertility preservation are currently very important. Most pregnancies after conservative treatment of BOT are spontaneous. However, not all studies differentiate spontaneous pregnancy from those obtained after fertility treatment. Some studies analysing the contribution of assisted reproductive technology (ART) are summarized in Table 3.

### Relation between BOT and infertility

Patients with BOT present a previous history of infertility in 10–35%, especially when BOT is serous, bilateral or with micropapillary pattern [18, 78]. Moreover, as discussed previously, surgery for BOT is also a cause of infertility due to adherences and the alteration of ovarian function. In patients with bilateral massive BOT, for which preservation of part of an ovary is not feasible, BSO should only be performed and the uterus should then be preserved, as pregnancies have been reported in patients who underwent BSO with uterine preservation using oocyte donation or a transfer of frozen embryos obtained before the BSO [82].

### Risk of BOT linked to hormonal fertility treatment

Little is known of the impact of fertility drugs after conservative treatment for BOT, but some authors have suggested a potential association between infertility, ovulation-inducing drugs, and BOT [18]. Rossing *et al* (2004) described ovarian tumours developed in women who had used clomiphene, representing a higher risk than that found for infertile women who had never used the drug. The risk of developing an ovarian tumour had increased for patients using clomiphene for 12 or more cycles [83]. In a nationwide study of women who received IVF treatment in the Netherlands, van Leeuwen *et al* (2011) also found that ovarian stimulation for IVF may increase the risk of ovarian malignancies, especially BOT [84]. In the study presented by Gotlieb *et al* (1998) most patients were diagnosed with BOT shortly afterwards ovulation induction or had received a few courses of ovulation-inducing drugs. Therefore, a causative correlation between borderline tumours and infertility treatment could not be established [18]. Basille *et al* (2006) reported no stimulatory effect of FSH or E2 on cell cultures from BOT, suggesting that gonadotrophins could safely be used in infertile patients after conservative surgery for BOT [85]. To analyse these results it is important to take into account that patients who are eligible for ART are more often diagnosed on early-stage, and have consequently a better prognosis.

Some authors have suggested that it would be appropriate to wait at least one or two years after fertility-sparing surgery of BOT before to procede with ART, due to the possibility of achieving a spontaneous pregnancy and because the risk of recurrence is higher during the two first post-operative years [76, 78]. However, a long period between surgery and ART has a potential negative impact on fertility outcome due to the deterioration of the ovarian reserve.

### In vitro fertilization

*In vitro* Fertilization (IVF) is a good option for infertility problems in patients previously diagnosed of BOT, due to the high pregnancy rate of this procedure and the fact that no adverse effect of IVF has been demonstrated in patients in the early stages [58, 86]. Fortin *et al* (2007) reported a pregnancy rate of 40% after IVF. They observed four recurrences over 25 patients but no death from the disease, and no intraperitoneal or vaginal dissemination occurred after oocyte collection [86]. However, there is not sufficient data about IVF in patients with advanced stages BOT and, therefore, we cannot exclude potential side effects. Fatemi *et al* (2011) reported the first case of ex vivo re-trieval of mature oocytes after ovarian stimulation via laparotomy, with the intention to avoid potential dissemination linked to oocyte retrieval [87]. Further studies are required to clarify the role of this technique compared with classic IVF.

### Ovarian cryopreservation

Cryopreservation of ovarian tissue is a potential option recommended by several teams [58, 88]. However, retransplantation of ovarian tissue exposes patients to the potential risk of transplantation of borderline cells [89].

Published studies of cryopreservation in patients with BOT still have a small number of patients. Fain-Kahn *et al* (2009) reported a series of 17 patients, cryopreservation of ovarian tissue was possible in 9 (53%), the remaining patients did not have normal ovarian parenchyma to perform this technique [88]. Ovarian tissue culture with in vitro maturation and follicle isolation have been described, where the metaphase I oocytes can be retrieved and matured in-vitro and then cryopreserved by vitrification. The oocyte vitrification has a post-thaw survival rate of 94% and a pregnancy rate of 46.7% [88, 90]. Cryopreservation of immature oocytes from fresh tissue or follicular aspirates is another option [90].

Sometimes the recurrence affects the entire remaining ovary, and a second conservative surgery is not feasible, so it is too late for ovarian cryopreservation. Donnez *et al* (1998) proposed cryopreservation of contralateral ovarian cortex biopsy at first-look laparoscopy if the borderline nature of the tumour is suspected before surgery. On the other hand, they recommend cortex biopsy in a second separate procedure after diagnosis of BOT before any possible relapse [91]. This procedure is an interesting solution for young women with early stage disease. Careful selection of candidates for this kind of treatment is, of course, necessary and close follow-up is required [20, 91].

### Radical fimbriectomy

Another option as an alternative to salpingo-oophorectomy, in view of the recent literature on ovarian carcinogenesis, would be radical fimbriectomy, especially for patients with advanced stage BOT and peritoneal implants. This procedure consists of removing all the tube and the fimbrio-ovarian junction, aimed to protect high-risk women from high-grade serous pelvic carcinoma, while preserving their ovarian function. The safety and validity of this procedure has not yet been confirmed by a multi-institutional study [92].

## Conclusions

In conclusion, conservative treatment of early stages BOT results in a high pregnancy rate and low recurrence rate as well as the low risk of death from the disease. USO should be considered as the first choice of fertility-sparing treatment for BOTs in women wishing to preserve fertility, providing a safe therapeutic treatment, and ovarian cystectomy should be considered for women with one ovary or with bilateral tumours who wish to preserve their childbearing potential, and who are willing to undergo careful and prolonged follow-up examinations. Comprehensive restaging has a minor effect on clinical management and outcome. No further radical surgery is generally needed, and very close follow-up should be enough. Patients with advanced-stage have an excellent prognosis if the tumour does not have invasive implants, therefore, fertility-sparing surgery should be considered for patient with a strong desire to become pregnant if there are no invasive implants.

## Conflicts of interest

The authors have no conflicts of interest to declare.

## Figures and Tables

**Table 1.** Published studies comparing the oncologic outcomes after fertility-sparing surgery for BOTs.

Authors	Patients (*n*)	Surgical procedure	Recurrence *n* (%)	Histology of recurrence	Interval to recurrence (mo)	Death (*n*)	F/u (mo)
Zanetta *et al* (2001)	189	C 50 USO 139	14 (28%) 21 (15.1%)	9-BOT, 5-IOC 20-BOT, 1-IOC	39 45	1	70
Song *et al* (2011)	155	C 38 USO 117	5 (13.2%) 7 (5.9%)	5-BOT 6-BOT, 1-IOC	28 42	0	56
Yinon *et al* (2007)	62	C 22 USO 40	5 (22.7%) 11 (27.5%)	5-BOT 10-BOT	23.6 41	0	88
Morice *et al* (2001)	44	C 11 USO 33	4 (36.3%) 5 (15.1%)	4-BOT 5-BOT	-	0	109
Park *et al* (2009)	184	C 56 USO 128	6 (10.7%) 3 (2.3%)	6-BOT 2-BOT, 1-IOC	10 49.5	1	70
Boran *et al* (2005)	62	C 22 USO 40	3 (13.6%) 1 (2.5%)	3 BOT 1 BOT	24 48	0	44.3
Donnez *et al* (2003)	16	C 5 USO 11	1 (20%) 2 (18.2%)	1 BOT 2 BOT	12 21	0	43.4
Seracchioli *et al* (2001)	19	C 11 USO 8	1 (9%) 0	1 BOT	6	0	42
Gotlieb *et al* (1998)	39	C 12 USO 27	2 (16.6%) 2 (7.4%)	2 BOT 1 BOT, 1 IOC	82.5 33	0	70
Camatte *et al* (2002)	68	C 21 USO 47	4 (19%) 5 (10.6%)	4 BOT 5 BOT	-	0	71
Longacre *et al* (2005)	53	-	9 (17%)^*^	7 BOT, 2 IOC^*^	-	0	60
Fauvet *et al* (2005)	162	-	27 (17%)^*^	27 BOT^*^	39^*^	0	-
Romagnolo *et al* (2006),	53	C 21 USO 32	6 (28.6%) 7 (21.9%)	6 BOT 7 BOT	-	1	44
Wong *et al* (2007)	116	C 38 USO 78	2 (5.3%) 2 (2.3%)	2 BOT, 2 IOC^*^	59^*^	1	21
Kanat-Pektas *et al* (2011)	55	C 19 USO 36	2 (10.5%) 1 (2.8%)	2 BOT 1 BOT	-	0	61
Khunamornpong *et al* (2011)	60	C 1 USO 59	6 (10%)^*^	2 BOT, 4 IOC^*^	-	2	-

C: Cystectomy

USO: Unilateral salpingo-oophorectomy

IOC: Invasive ovarian cancer

* Data is not divided by type of surgery

**Table 2.** Published studies comparing pregnancy outcomes after fertility-sparing surgery for BOTs.

Authors	Patients (*n*)	Desire for pregnancy (*n*)	Pregnancy (*n*)	Total pregnancies	Pregnancy rate (%)	Term babys	Interval^*^ (mo)
Song *et al* (2011)	155	51	45	58	88.2	54, 4 ongoing	28
Yinon *et al* (2007)	62	–	25	38	40.3	35	–
Morice *et al* (2001)	44	–	14	17	31.8	–	–
Park *et al* (2009)	184	31	27	–	87.1	34	–
Boran *et al* (2005)	62	25	10	13	40	10	13.6
Donnez *et al* (2003)	16	11	7	12	63.6	12	–
Seracchioli *et al* (2001)	19	10	6	6	60	6	–
Gotlieb *et al* (1998)	39	–	15	22	38.5	19, 3 ongoing	30
Fauvet *et al* (2005)	162	65	21	30	32.3	–	–
Kanat-Pektas *et al* (2011)	55	44	23	23	52.3	–	–
Romagnolo *et al* (2006)	53	12	7	8	58.3	7	–

* Interval to pregnancy since surgery for BOT.

**Table 3.** Results of Assisted Reproductive Technology (ART) in BOTs (for early and advanced stage).

Authors	Patients (*n*)	Ovulation Induction^*^	IVF/ICSI	Pregnancy (*n*)	Total pregnancies	Pregnancy rate (%)	Recurrences after stimulation (*n*)
Palomba *et al* (2010)	32	29	26	24	48	75	20
Yinon *et al* (2007)	5	0	5	5	6	100	1
Morris *et al* (2000)	6	4	2	4	–	66.7	–
Gallot *et al* (2000)	1	0	1	1	–	100	0
Gotlieb *et al* (1998)	11	11	0	4	–	36.4	0
Camatte *et al* (2002)	5	1	4	2	2	40	2
Fauvet *et al* (2005)	11	6	5	3	3	27.3	0
Fortin *et al* (2007)	30	3	27	13	13	43.3	4
Song *et al* (2011)	5	3	2	5	–	100	–

* Ovulation Induction with clomiphene
